# Complete chloroplast genome sequence of *Bambusa rigida* (Bambuseae)

**DOI:** 10.1080/23802359.2020.1793699

**Published:** 2020-07-23

**Authors:** Ying Zheng, Dan Hou, Juan Zhuo, Renhong Zheng, Yong Wang, Benxiang Li, Xuejun Yu, Xinchun Lin

**Affiliations:** a State Key Laboratory of Subtropical Silviculture, Zhejiang A&F University, Hangzhou, Zhejiang, P. R. China; b Yibin Forestry and Bamboo Industry Research Institute, Yibin, Sichuan, P. R. China; c Sichuan Changning Century Bamboo Garden, Yibin, Sichuan, P. R. China

**Keywords:** *Bambusa rigida*, chloroplast genome, phylogeny

## Abstract

*Bambusa rigida* is a tropical woody bamboo widely distributed in Sichuan and southeastern of China with important economic and ecological values. We performed a complete chloroplast (cp) genome of *B*. *rigida* using Illumina NovaSeq PE150 platform. The length of complete cp sequence is 139,500 bp in size with a pair of inverted repeat (IR) regions of 43,587 bp, which separates a large single-copy (LSC) region of 83,036 bp and a small single-copy (SSC) region of 12,875 bp. Plastid genome contains 132 genes, including 84 protein-coding genes, 40 tRNA genes, and 8 rRNA genes. Phylogenetic analysis based on 30 cp genomes indicates that *B. rigida* is closely related to *Bambusa ventricosa* in Bambuseae.

*Bambusa rigida* Keng et Keng f. is a tropical woody bamboo belonging to the family Gramineae (Bambusoideae: Bambuseae) with the rhizome pachymorph without enlonged necks (Li et al. 2006). It is widely distributed in Sichuan, Guizhou, Guangxi, Guangdong, Fujian provinces of China with important economic benefits (Liu et al. 2013) and ecological functions (P. Zhang et al. 2009). *Bambusa rigida* is a typical medium-sized sympodial bamboo species characterized by high stalk, thick wall, hard material, heavy weight, relatively small diameter, which made it a perfect raw material for paper making as well as for weaving, scaffolding, fence and garden greening (W. Zhang et al. 2014; Cao et al. 2018). In this study, we reported the complete chloroplast (cp) genome of *B. rigida* based on Illumina pair-end sequencing data.

Fresh and healthy leaves of *B. rigida* were collected from Yibin, Sichuan province (28°45′28″N, 104°38′34″E), and dried immediately by silica gel. The voucher specimen was kept at the College of Forestry and Biotechnology, Zhejiang A&F University (Accession No. BR01). Total genomic DNA was extracted using a modified CTAB protocol (Doyle 1991). Pair-end library was constructed and sequenced with Illumina NovaSeq PE150 platform (Novogene Bioinformatics Technology Co., Ltd in Beijing, China). The chloroplast genome of *B. rigida* was assembled by MITObim v1.8 (Hahn et al. 2013) and annotated with the PGA (Qu et al. 2019).

The complete chloroplast genome of *B. rigida* (GenBank accession: MT648824) was 139,500 bp in size with a large single-copy (LSC) region of 83,036 bp, a small single-copy (SSC) region of 12,875 bp, and a pair of inverted repeat (IR) regions of 43,587 bp. A total of 132 genes was found in the *B. rigida* chloroplast genome, including 84 protein-coding genes, 40 tRNA genes, and 8 rRNA genes. The complete genome GC content was 39.42%.

To reveal the phylogenetic relationship of *B. rigida* within the Gramineae family, a phylogenetic analysis was performed based on 28 complete chloroplast genomes of Bambusoideae with *Acidosasa purpurea* and *Ampelocalamus actinotrichus* as outgroups. All of the cp genome data were downloaded from NCBI GenBank database. The sequences were aligned by MAFFT (Katoh and Standley 2013). Phylogenetic analysis was conducted by RAxML-NG (Kozlov et al. 2019). The bootstrap values were calculated based on 1000 replicates. The phylogenetic tree revealed that *B. rigida* was most closely related to *Bambusa ventricosa* with strong support (Figure 1).

**Figure 1.**
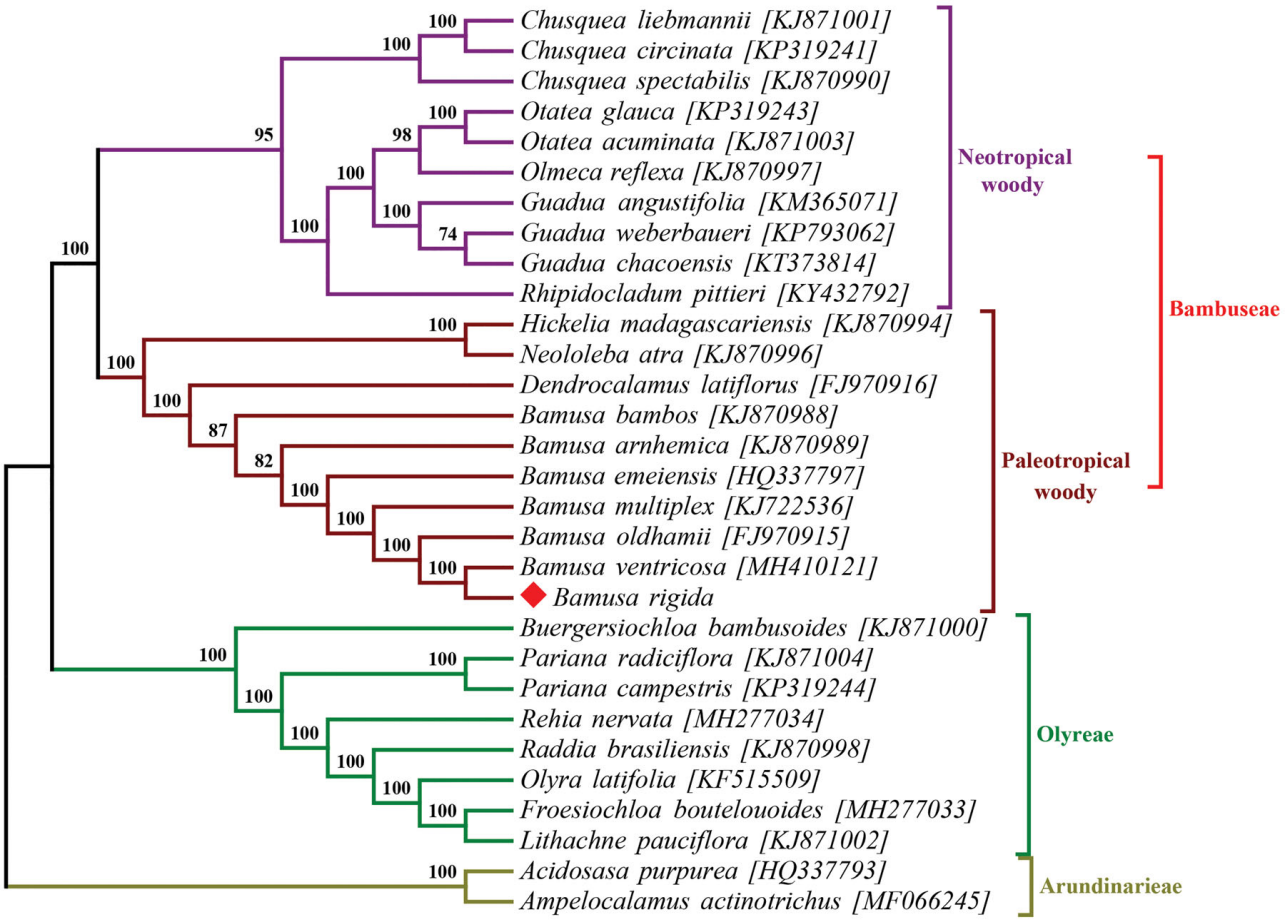
Maximum likelihood tree inferred from chloroplast genomes of 28 Bambusodae and two Arundinarodae (*Acidosasa purpurea* and *Ampelocalamus actinotrichus* as outgroup) by RAxML. Bootstrap support values were list on the nodes.

## Data Availability

The data that support the findings of this study have been deposited in the NCBI database [GenBank accession: MT648824] (https://www.ncbi.nlm.nih.gov/).

## References

[CIT0001] Cao Y, Zeng Y, Bie P, Zhao R, Chen J, Chen X. 2018. A study of the growth of *Bambusa rigida* in Changning County of Sichuan Province. J Sichuan Forest Sci Technol. 39:120–124.

[CIT0002] Doyle J. 1991. DNA protocols for plants. In: Hewitt GM, editor. Molecular techniques in taxonomy. Berlin: Springer-Verlag; p. 283–293.

[CIT0003] Hahn C, Bachmann L, Chevreux B. 2013. Reconstructing mitochondrial genomes directly from genomic next-generation sequencing reads – a baiting and iterative mapping approach. Nucleic Acids Res. 41(13):e129. 23661685 10.1093/nar/gkt371 PMC3711436

[CIT0004] Katoh K, Standley DM. 2013. MAFFT multiple sequence alignment software version 7: improvements in performance and usability. Mol Biol Evol. 30(4):772–780. 23329690 10.1093/molbev/mst010 PMC3603318

[CIT0005] Kozlov AM, Darriba D, Flouri T, Morel B, Stamatakis A. 2019. RAxML-NG: a fast, scalable and user-friendly tool for maximum likelihood phylogenetic inference. Bioinformatics. 35(21):4453–4455. 31070718 10.1093/bioinformatics/btz305 PMC6821337

[CIT0006] Li DZ, Wang ZP, Zhu ZD, Xia NH, Jia LZ, Guo ZH, Yang GY, Stapleton C. 2006. Bambuseae (Poaceae). In: Wu ZY, Raven PH, Hong DY, editors. Flora of China. Beijing and St. Louis: Science Press and Missouri Botanical Garden Press; p. 7–180.

[CIT0007] Liu G, Fan S, Cai C, Zhang D. 2013. Comparative Analysis on the Clonal Growth Characteristics of *Bambusa pervariabilis* × *Dendrocalamopsis daii* and *B. rigida*. Chin Bull Bot. 48:288–294.

[CIT0008] Qu XJ, Moore MJ, Li DZ, Yi TS. 2019. PGA: a software package for rapid, accurate, and flexible batch annotation of plastomes. Plant Methods. 15:50 31139240 10.1186/s13007-019-0435-7 PMC6528300

[CIT0009] Zhang P, Zhang X, Huang L, Liu W, Zhu W, Tang S. 2009. Interception effect of surface runoff on different width *Bambusa rigida* Keng Riparian Buffer Strips. J Soil Water Conserv. 23:23–27.

[CIT0010] Zhang W, Liu S, Tu S, Jiang B, Wu Z, Hu D, Tu S, Guo X. 2014. Culm form characteristics of *Bambusa rigida* and the influence of special bamboo fertilizer. J West China Forest Sci. 44:9–14.

